# Dibromo-Edaravone Induces Anti-Erythroleukemia Effects via the JAK2-STAT3 Signaling Pathway

**DOI:** 10.3390/ijms26094000

**Published:** 2025-04-23

**Authors:** Qiqing Chen, Sheng Liu, Xuenai Wei, Peng Zhao, Fen Tian, Kang Yang, Jingrui Song, Yubing Huang, Min Wen, Jialei Song, Yong Jian, Yanmei Li

**Affiliations:** 1 State Key Laboratory of Discovery and Utilization of Functional Components in Traditional Chinese Medicine, Guizhou Medical University, Guiyang 550014, China; 2022110030778@stu.gmc.edu.cn (Q.C.); liusheng@gmc.edu.cn (S.L.); 2024010030093@stu.gmc.edu.cn (X.W.); 2023010030139@stu.gmc.edu.cn (P.Z.); 2023110030655@stu.gmc.edu.cn (F.T.); 2023120030783@stu.gmc.edu.cn (K.Y.); songjingrui123@snnu.edu.cn (J.S.); huangyubing@gmc.edu.cn (Y.H.); 2 Natural Products Research Center of Guizhou Province, Guiyang 550014, China; 3 College of Pharmacy, Guizhou Medical University, Guiyang 550004, China; wenmin@gmc.edu.cn; 4 School of Basic Medicine, Guizhou University of Traditional Chinese Medicine, Guiyang 550025, China; songjialei083@gzy.edu.cn

**Keywords:** dibromo-edaravone, erythroleukemia, apoptosis, JAK2-STAT3, cell cycle

## Abstract

Acute erythroid leukemia (AEL) is a rare and aggressive hematological malignancy managed with chemotherapy, targeted therapies, and stem cell transplantation. However, these treatments often suffer from limitations such as refractoriness, high toxicity, recurrence, and drug resistance, underscoring the urgent need for novel therapeutic approaches. Dibromo-edaravone (D-EDA) is a synthetic derivative of edaravone (EDA) with unreported anti-leukemic properties. In this study, D-EDA demonstrated potent cytotoxicity against HEL cells with an IC_50_ value of 8.17 ± 0.43 μM using an MTT assay. Morphological analysis via inverted microscopy revealed reductions in cell number and signs of cellular crumpling and fragmentation. Flow cytometry analysis, Hoechst 33258 staining, Giemsa staining, a JC-1 assay, and a reactive oxygen species (ROS) assay showed that D-EDA induced apoptosis in HEL cells. Furthermore, D-EDA induced S-phase cell cycle arrest. Western blot analysis showed significant upregulation of key apoptosis-related proteins, including cleaved caspase-9, cleaved caspase-3, and cleaved poly ADP-ribose polymerase (PARP), alongside a reduction in Bcl-2 expression. Additionally, oncogenic markers such as c-Myc, CyclinA2, and CDK2 were downregulated, while the cell cycle inhibitor p21 was upregulated. Mechanistic studies involving molecular docking, a cellular thermal shift assay (CETSA), the caspase inhibitor Z-VAD-FMK, JAK2 inhibitor Ruxolitinib, and STAT3 inhibitor Stattic revealed that D-EDA activates the caspase cascade and inhibits the JAK2-STAT3 signaling pathway in HEL cells. In vivo, D-EDA improved spleen structure, increased the hemolysis ratio, and extended survival in a mouse model of acute erythroleukemia. In conclusion, D-EDA induces apoptosis via the caspase cascade and JAK2-STAT3 signaling pathway, demonstrating significant anti-leukemia effects in vitro and in vivo. Thus, D-EDA may be developed as a potential therapeutic agent for acute erythroleukemia.

## 1. Introduction

Myeloma and lymphoma, collectively known as hematological malignancies, arise from disruptions in normal hematopoiesis and remain a significant global health challenge. These malignancies include leukemia, multiple myeloma (MM), non-Hodgkin’s lymphoma (NHL), and Hodgkin’s lymphoma (HL) [1]. Acute erythroleukemia (AEL), a rare and aggressive subtype of acute myeloid leukemia (AML), accounts for 2% of AML cases. The aggressive nature, rarity, and high clinical risk associated with AEL make it particularly difficult to treat, often resulting in poor clinical outcomes [2,3,4,5]. Current first-line treatments for AEL involve intensive chemotherapy and hypomethylating agents; however, these approaches are limited by issues such as drug resistance, high toxicity, recurrence, and refractory disease. In addition, allogeneic bone marrow transplantation and targeted therapies have advanced in the treatment of leukemia. However, these approaches remain challenging and limited in their effectiveness for AEL patients [2,6]. Consequently, there is an urgent need to identify practical, low-toxicity therapeutic agents and optimize treatment regimens to improve survival rates and quality of life for patients with AEL.

Edaravone (EDA) is a known free radical scavenger that inhibits lipid peroxidation and is currently utilized in the treatment of cerebral infarction [7] and amyotrophic lateral sclerosis [8]. Additionally, research indicates that EDA may alleviate symptoms of depression and anxiety-like behavior [9]. Notably, EDA exhibits antioxidant and anti-inflammatory properties, which could potentially be harnessed in cancer treatment [10]. Dibromo-edaravone (D-EDA) is a derivative of edaravone, created through structural modifications. However, the role and mechanism of D-EDA in leukemia treatment have yet to be explored.

The JAK-STAT pathway is an evolutionarily conserved transmembrane signal transduction mechanism and one of the central communication nodes in cellular function. This pathway enables the transmission of extracellular signals from the cell membrane to the nucleus, playing a pivotal role in essential physiological processes such as hematopoiesis, differentiation, metabolism, and immune regulation. However, aberrant activation of JAK-STAT signaling induces many diseases, especially immune-related diseases and cancers [11,12,13,14]. Given its crucial role in disease, the JAK-STAT pathway is a significant target for leukemia therapy. The JAK-STAT signaling pathway describes how cytokines bind to specific receptors, activating the associated JAK kinase. The activated JAK then phosphorylates a site on the receptor, recruiting and phosphorylating STAT proteins. These STAT proteins subsequently dimerize and translocate to the nucleus, where they regulate the transcription of target genes. The JAK family consists of four members: JAK1, JAK2, JAK3, and TYK2 [15,16]. The STAT family comprises seven members: STAT1, STAT2, STAT3, STAT4, STAT5a, STAT5b, and STAT6 [17]. Mutations in JAK-STAT pathway genes have been identified as key drivers of cancer. Among these, somatic gain-of-function mutations in the JAK genes are frequently linked to hematologic malignancies. For instance, JAK2 mutations are a hallmark of myeloproliferative disorders, including polycythemia vera, essential thrombocythemia, and primary myelofibrosis. Similarly, STAT3 mutations, the most common STAT mutations in cancer, are prevalent in hematologic tumors [18,19,20].

In this study, we evaluated the effects of D-EDA on the biological functions of HEL cells using MTT assay, apoptosis, and cell cycle assays in vitro. Furthermore, Western blot and cellular thermal shift assays further analyzed the potential mechanism. Finally, we assessed the therapeutic effects of D-EDA in a mouse model of acute erythroleukemia.

## 2. Results

### 2.1. D-EDA Inhibits the Viability of HEL Cells

**Figure 1:**
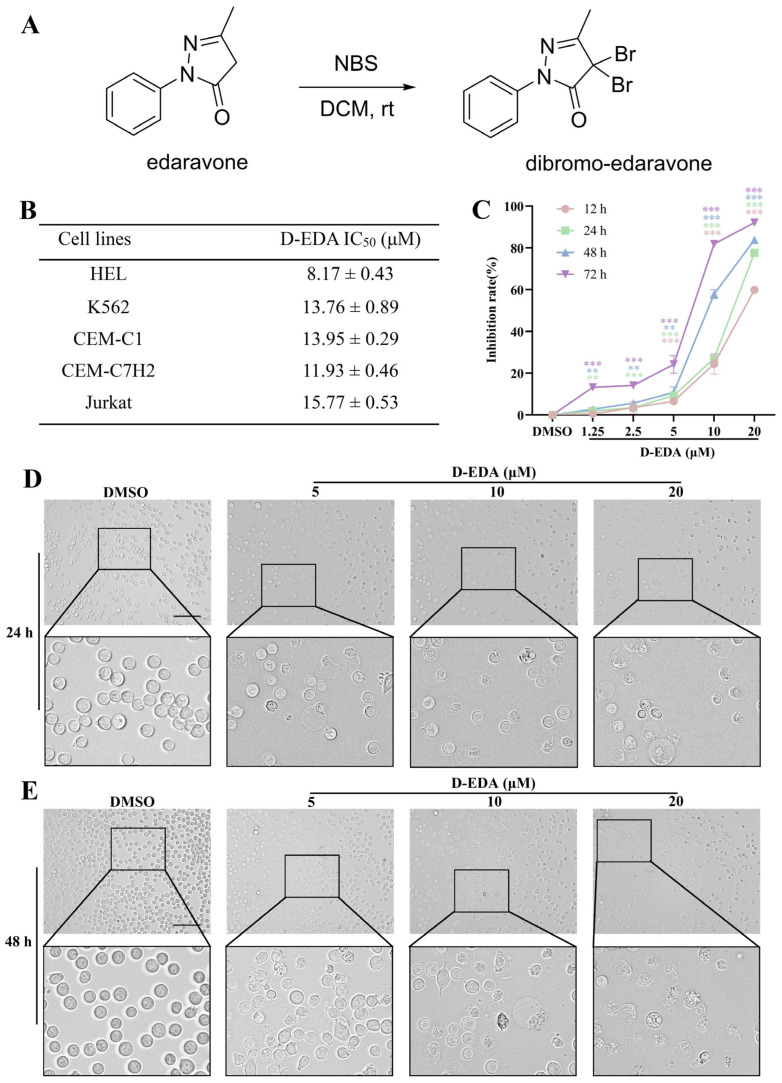
D-EDA inhibits HEL cell viability. (**A**) Chemical structure of D-EDA. (**B**) IC_50_ values of various leukemia cell lines treated with D-EDA for 48 h. (**C**) Growth curves of HEL cells treated with D-EDA for 12, 24, 48, and 72 h. (**D**,**E**) Morphological changes of D-EDA-interacting HEL cells at 24 h, and 48 h (Magnification: 200×, Scale bar: 100 μm). Data are denoted as mean ± SD (*n* = 3. ** *p* < 0.01, *** *p* < 0.001 vs. the control group).

Edaravone (EDA) is a well-known free radical scavenger that inhibits lipid peroxidation and is currently utilized in the treatment of cerebral infarction [7]. Dibromo-edaravone (D-EDA) is a novel derivative of EDA, developed through structural modifications (Figure 1A). To investigate the effects of D-EDA on various leukemia cell lines, including HEL, Jurkat, CEM-C1, and CEM-C7H2, we treated these cells with different concentrations of D-EDA for 48 h. The results indicated that D-EDA significantly affected these cell lines, yielding IC_50_ values of 8.17 ± 0.43 μM for HEL, 13.76 ± 0.89 μM for K562, 13.95 ± 0.29 μM for CEM-C1, and 11.93 ± 0.46 μM for CEM-C7H2, as well as 15.77 ± 0.53 μM for Jurkat, respectively. Notably, D-EDA had the most pronounced effect on HEL cells (Figure 1B). The treatment with varying concentrations of D-EDA over 12, 24, 48, and 72 h significantly inhibited the viability of HEL cells in a concentration- and time-dependent manner (Figure 1C). Further analysis revealed that EDA did not impact HEL cell viability; specifically, the survival rate of HEL cells treated with EDA (20 μM) for 72 h was 100.65 ± 0.052% (Supplementary Figure S1). Additionally, to compare the effects of D-EDA with Ruxolitinib on HEL cells, we performed an MTT assay to assess cell viability after 24 or 48 h of treatment. The results indicated that the inhibition rates of HEL cells treated with Ruxolitinib (20 μM) or D-EDA (20 μM) for 48 h were 46.02 ± 0.339% and 83.88 ± 0.003%, respectively, indicating that D-EDA exhibited a greater inhibition rate than Ruxolitinib after 48 h (Supplementary Figure S3). Moreover, microscopic evaluation revealed that treatment with D-EDA led to a decrease in cell numbers, along with signs of crumpling and fragmentation of HEL cells after both 24 h (Figure 1D) and 48 h (Figure 1E) of treatment.

### 2.2. D-EDA Induces Apoptosis in HEL Cells

**Figure 2:**
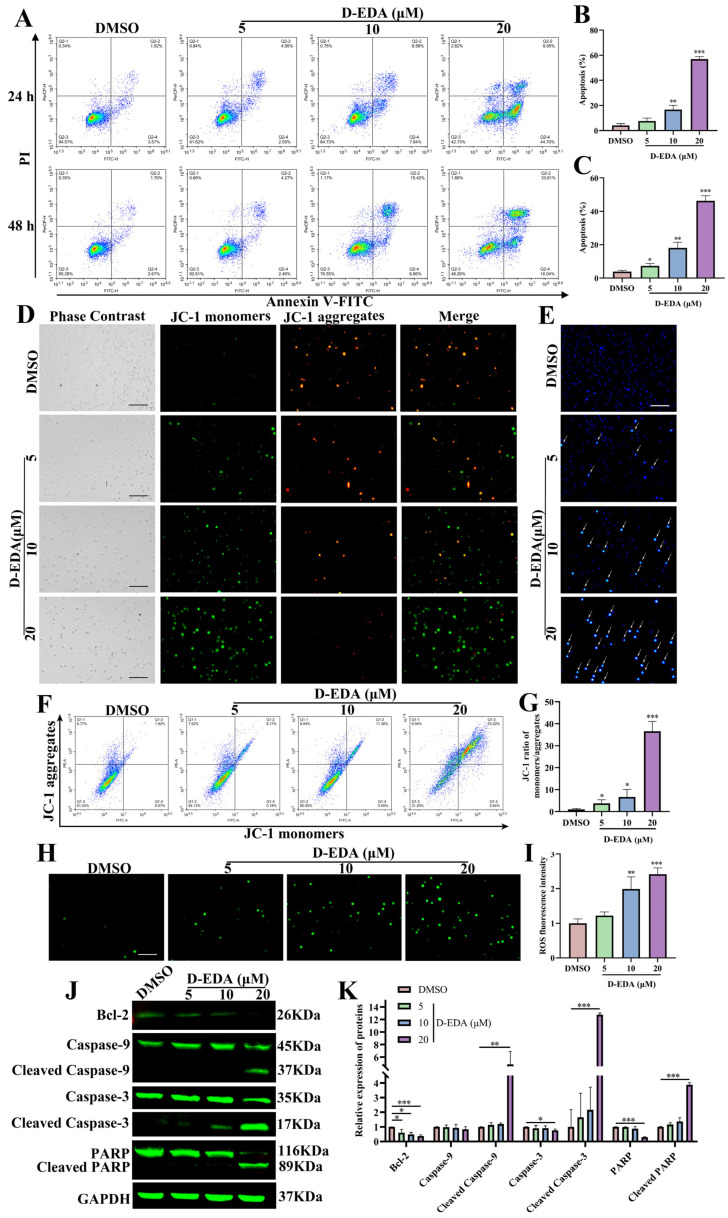
D-EDA induces apoptosis in HEL cells. (**A**) The impact of D-EDA on HEL cell apoptosis was analyzed by flow cytometry after 24 h and 48 h. (**B**,**C**) Statistical graph of apoptosis. (**D**) The effect of D-EDA on the MMP of HEL cells at 24 h (The green fluorescence indicates JC-1 monomers, and the red fluorescence indicates JC-1 aggregates.). (**E**) The effect of D-EDA on Hoechst 33258 in HEL cells after 48 h of treatment. (**F**) Changes in MMP in HEL cells affected by D-EDA were detected by flow cytometry. (**G**) A statistical graph of MMP. (**H**) Fluorescence inversion microscopy was used to detect the effect of D-EDA on 24 h ROS in HEL cells. (**I**) Statistical graph of changes in ROS detected by flow cytometry. (**J**) The impact of D-EDA on the expression of apoptotic proteins Bcl-2, Caspase-9, Caspase-3, PARP, Cleaved Caspase-9, Cleaved Caspase-3, and Cleaved PARP in HEL cells was detected by Western blot. (**K**) Statistical graph of apoptotic proteins expression (Magnification: 200×, Scale bar: 100 μm). Data are denoted as mean ± SD (*n* = 3. * *p* < 0.05, ** *p* < 0.01, *** *p* < 0.001 vs. the control group).

Apoptosis, the most noted form of programmed cell death, manipulates a key physiological mechanism that impairs cell viability [21]. To determine whether the inhibition of HEL cell viability by D-EDA was due to apoptosis, we assessed apoptosis through flow cytometry. The results demonstrated that D-EDA significantly induced apoptosis in HEL cells in a dose-dependent manner compared to the DMSO group. At a concentration of 20 μM, the apoptosis rate at 24 h was 56.90 ± 1.979%, predominantly occurring in the early apoptotic phase. At 48 h, the apoptosis rate was 46.28 ± 3.097%, primarily reflecting late apoptosis (Figure 2A–C). Observations made using the Hoechst 33258 assay and fluorescence microscopy revealed densely packed nuclei with increased blue-white fluorescence (indicated by arrowheads) compared to the DMSO group, suggesting that D-EDA may induce DNA damage in the cell nuclei (Figure 2E). Further analysis via Giemsa staining showed that apoptotic cells exhibited characteristic morphological changes, including nuclear fragmentation, ruffled cell membranes, membrane curling, and vesicle formation (Supplementary Figure S2). Western blot analysis indicated that D-EDA reduced the protein expression levels of Bcl-2 while increasing the levels of Cleaved Caspase-9, Cleaved Caspase-3, and Cleaved PARP proteins, with the most pronounced effects observed at a concentration of 20 μM (Figure 2J,K).

### 2.3. D-EDA Reduces HEL Cells’ MMP and Increases ROS Levels in HEL Cells

MMP is a critical biological event in the early stages of apoptosis, and elevated levels of reactive oxygen species (ROS) can result in mitochondrial damage [22]. To elucidate the mechanism of apoptosis induced by D-EDA, we employed JC-1 staining and ROS assays to analyze changes in MMP and ROS in HEL cells treated with D-EDA, utilizing fluorescence microscopy and flow cytometry. The results indicated that, with increasing concentrations of D-EDA, green fluorescence gradually increased while red fluorescence decreased compared to the DMSO group. This study suggests that D-EDA down-regulates MMP in HEL cells in a concentration-dependent manner (Figure 2D,F,G). Furthermore, ROS levels in HEL cells were significantly elevated as the concentration of D-EDA increased relative to the DMSO group (Figure 2H,I). These results indicate that D-EDA inhibits the growth of HEL cells through apoptosis, which may be associated with the loss of MMP and increased ROS levels.

### 2.4. D-EDA Induces Apoptosis by Activating the Caspase Cascade Reaction and Blocking the S Phase Cycle in HEL Cells

**Figure 3:**
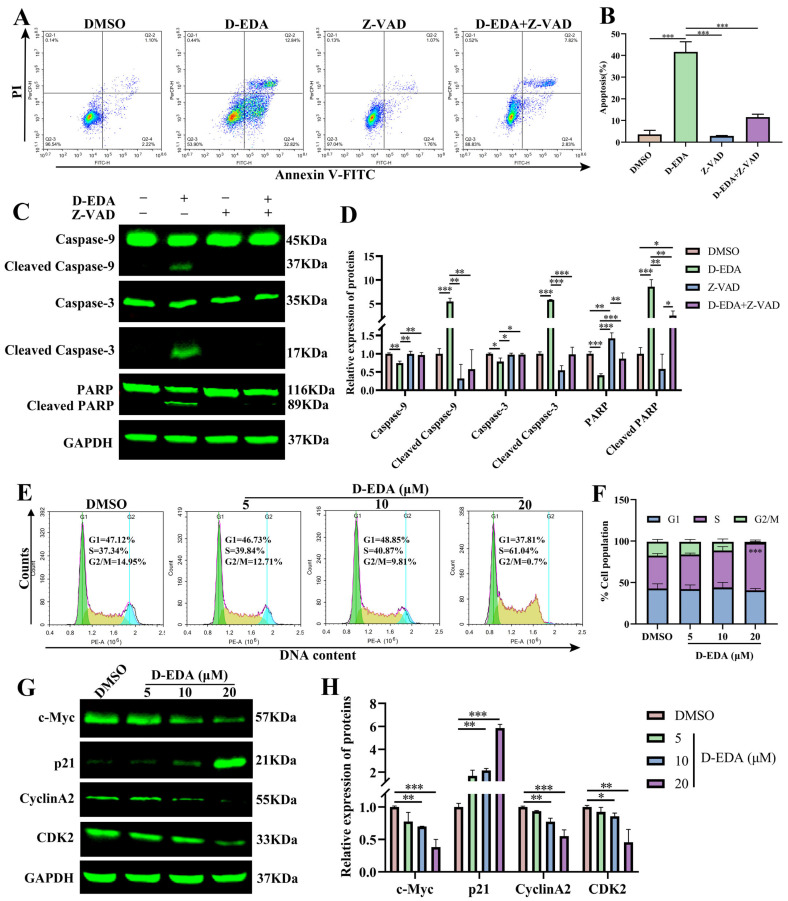
D-EDA activated the caspase cascade reaction and blocked the S phase in HEL cells. (**A**) The effect of Z-VAD on apoptosis of HEL cells at 24 h was detected by flow cytometry. (**B**) Apoptosis statistics graph. (**C**) Protein expression changes of Caspase-9, Caspase-3, PARP, Cleaved Caspase-9, Cleaved Caspase-3 and Cleaved PARP. (**D**) Statistical graph of expression of apoptotic proteins. (**E**) The impact of D-EDA on the cell cycle of HEL cells was analyzed after 24 h using flow cytometry (G1 phase: green; S phase: yellow; G2/M phase: blue). (**F**) Cell cycle statistics. (**G**) Effects of D-EDA on HEL cells for 24 h on cycle proteins: c-Myc, p21, CyclinA2 and CDK2. (**H**) Statistical chart of cell cycle proteins. Data are denoted as mean ± SD (*n* = 3. * *p* < 0.05, ** *p* < 0.01, *** *p* < 0.001).

Pro-apoptotic signal transducing cascades are initiated by MMP [23]. To determine whether D-EDA triggers apoptosis via the caspase cascade reaction, HEL cells were treated with Z-VAD-FMK alone or in combination with D-EDA for 24 h. Flow cytometry results indicated that the combination of Z-VAD (50 μM) and D-EDA (20 μM) significantly reduced the apoptosis rate of HEL cells compared to treatment with D-EDA alone (Figure 3A,B). Additionally, Western blot analysis demonstrated that this combination significantly decreased the levels of Cleaved Caspase-9, Cleaved Caspase-3, and Cleaved PARP proteins (Figure 3C,D). To further investigate the effects of D-EDA on the HEL cell cycle, HEL cells were treated with different concentrations of D-EDA for 24 h. The results revealed that D-EDA arrested the S phase of HEL cells in a dose-dependent manner (Figure 3E,F). Additionally, D-EDA reduced the protein levels of c-Myc, CyclinA2, and CDK2 in HEL cells while increasing the protein level of p21 (Figure 3G,H). These results indicated that D-EDA suppresses HEL cells’ viability by inducing apoptosis by activating the caspase cascade and blocking the cell cycle.

### 2.5. D-EDA Regulates the JAK2-STAT3 Signaling Pathway in HEL Cells

**Figure 4:**
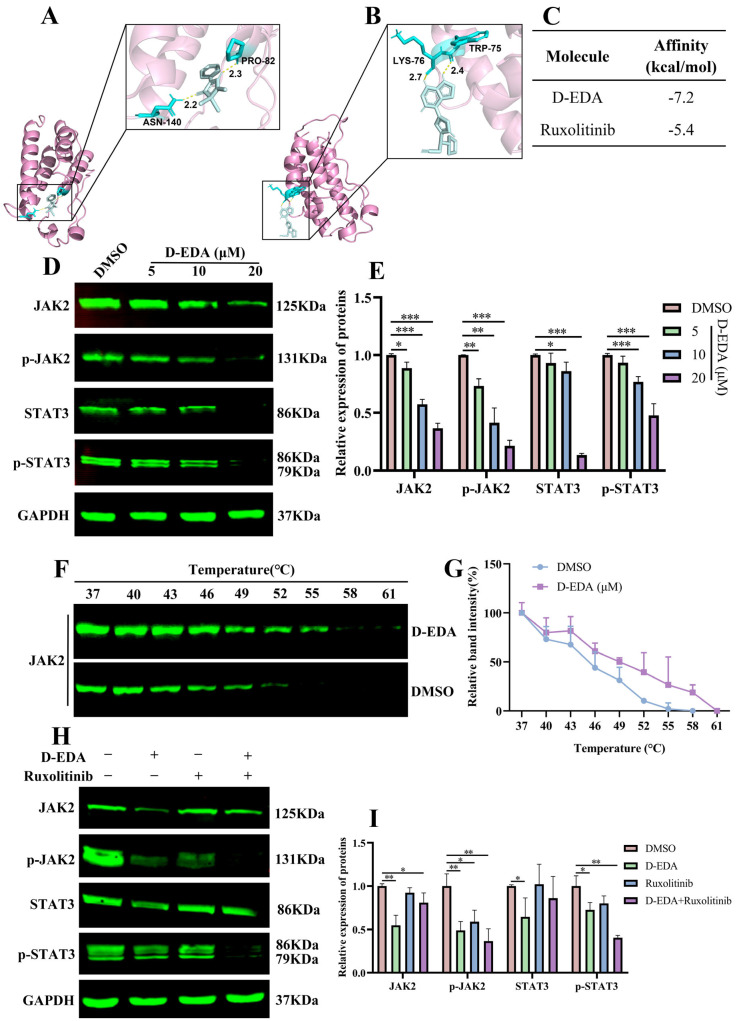
D-EDA inhibits the JAK2-STAT3 signaling pathway in HEL cells. (**A**–**C**) To predict the binding ability of JAK2 to D-EDA and Ruxolitinib by molecular docking assay. (**D**,**E**) Protein expression changes and statistical plots of JAK2, STAT3, p-JAK2, and p-STAT3. (**F**) Cellular thermal shift assay to detect the binding stability of D-EDA to JAK2. (**G**) Graph of thermal fusion curves of JAK2. (**H**,**I**) The impact of Ruxolitinib combined with D-EDA on JAK2, p-JAK2, STAT3, and p-STAT3 protein expression, along with statistical plots. Data are denoted as mean ± SD (*n* = 3. * *p* < 0.05, ** *p* < 0.01, *** *p* < 0.001 vs. the control group).

The JAK2/STAT3 signaling pathway plays a vital role in cell apoptosis [24]. To evaluate the impact of D-EDA on the JAK2/STAT3 signaling pathway, we employed Western blot analysis to assess the levels of related proteins in HEL cells following D-EDA treatment. The results revealed that D-EDA significantly down-regulated the protein levels of phosphorylated JAK2 (p-JAK2) and phosphorylated STAT3 (p-STAT3), with the most pronounced effects observed at a concentration of 20 μM (Figure 4D,E). Additionally, we examined JAK2 expression at the mRNA level in D-EDA-treated HEL cells using quantitative PCR (qPCR). The findings indicated that D-EDA did not affect JAK2 mRNA level (Supplementary Figure S4).

**Figure 5:**
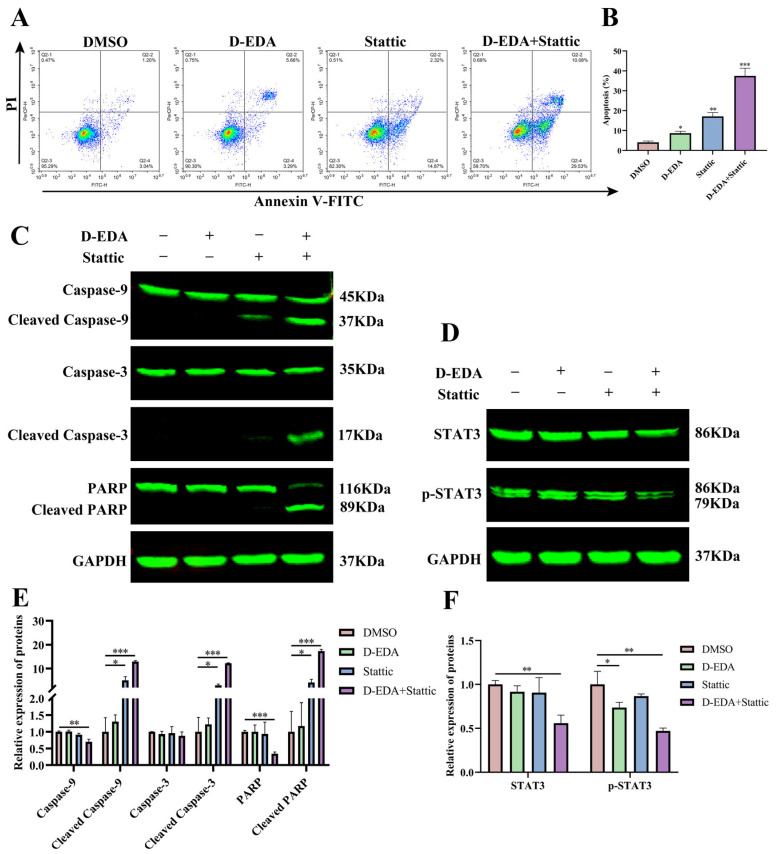
The combination of Stattic and D-EDA exhibits an anti-erythroleukemia effect by activating the caspase cascade reaction. (**A**) The effect of Stattic in combination with D-EDA on HEL cell apoptosis at 24 h was examined by flow cytometry. (**B**) Graph of apoptosis statistics. (**C**,**E**) Effect of Stattic with D-EDA on the expression of Caspase-9, Caspase-3, PARP, Cleaved Caspase-9, Cleaved Caspase-3, and Cleaved PARP proteins and statistical analyses. (**D**,**F**) The impact of Stattic combined with D-EDA on STAT3 and p-STAT3 protein expression, along with statistical plots. Data are denoted as mean ± SD (*n* = 3. * *p* < 0.05, ** *p* < 0.01, *** *p* < 0.001 vs. the control group).

To further investigate the target genes of D-EDA, we initially utilized molecular docking to predict its interaction with JAK2, which was subsequently validated using cellular thermal shift assays and inhibitors. The molecular docking results indicated that the binding energy of D-EDA to JAK2 was −7.2 kcal/mol (Figure 4A,C), while Ruxolitinib exhibited a binding energy of −5.4 kcal/mol (Figure 4B,C). To confirm whether D-EDA exerts its effects on HEL cells through the JAK2/STAT3 signaling pathway, we applied the JAK2 inhibitor Ruxolitinib to HEL cells. Western blot analysis demonstrated that the combination of D-EDA (10 μM) and Ruxolitinib (5 nM) significantly reduced the levels of p-JAK2 and p-STAT3 proteins compared to D-EDA alone (Figure 4H,I). The stability of D-EDA binding to JAK2 was further confirmed through cellular thermal shift assays (CETSA), which showed that the thermal degradation of D-EDA in complex with JAK2 was slower and exhibited greater stability compared to the DMSO group (Figure 4F,G). Further investigation of HEL cells using Stattic, a STAT3 inhibitor, revealed that the combination of D-EDA (10 μM) and Stattic (4 μM) significantly induced apoptosis compared to the DMSO group (Figure 5A,B). Flow cytometry results indicated a marked increase in apoptotic cells. Western blot analysis demonstrated that this combination significantly up-regulated the protein levels of Cleaved Caspase-9, Cleaved Caspase-3, and Cleaved PARP (Figure 5C,E), while significantly down-regulating the expression of p-STAT3 (Figure 5D,F). Moreover, JAK2 plays a key role in K562 proliferation [25]. We utilized Western blot analysis to investigate whether D-EDA affected p-JAK2 protein expression in K562 cells. The results confirmed that D-EDA also down-regulated the protein level of p-JAK2 (Supplementary Figure S5).

### 2.6. D-EDA Effectively Treats Mice with Acute Erythroleukemia

**Figure 6:**
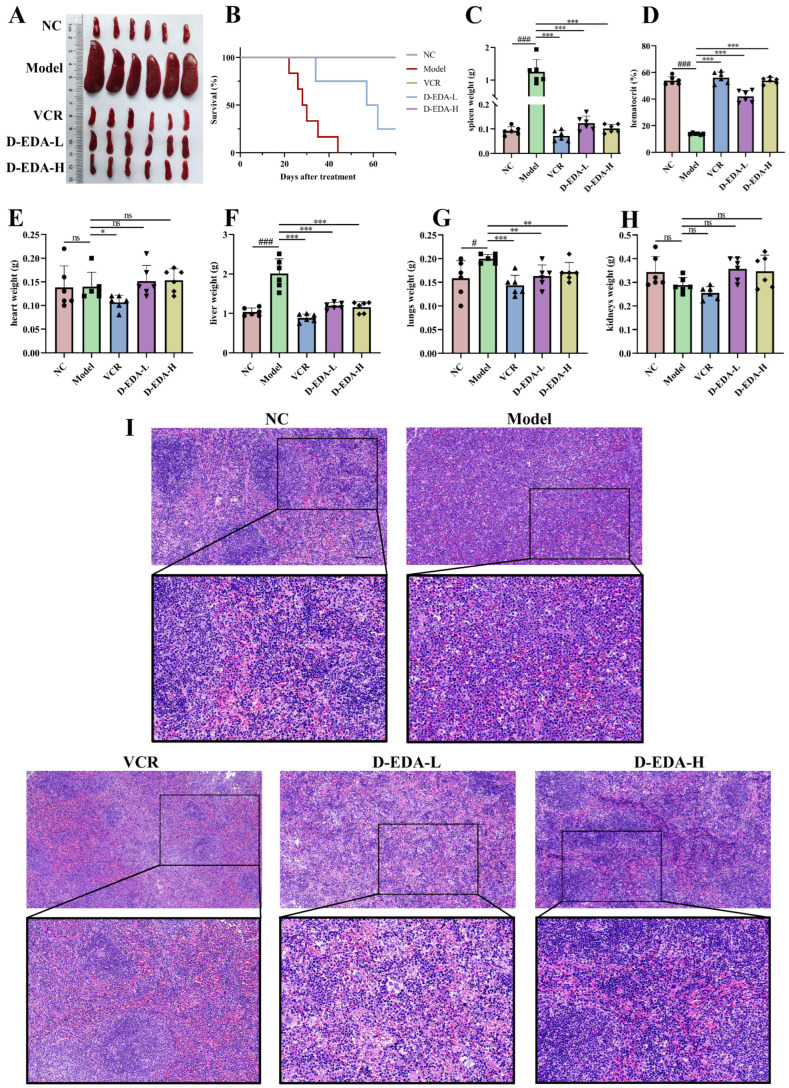
D-EDA blocks the progress of F-MuLV-induced erythroleukemia in mice. (**A**) Photographs of the spleens of mice in each group (*n* = 6). (**B**) Spleen weight statistics of mice. (**C**) Hematocrit statistics of mice. (**D**) Heart weight statistics of mice. (**E**) Liver weight statistics of mice. (**F**) Weight statistics of mice lungs. (**G**) Weight statistics of mice kidneys. (**H**) Growth curves after D-EDA treatment in mice (*n* = 6). (**I**) H&E staining of the spleen (Magnification: 200×, Scale bar: 100 μm). Data are denoted as mean ± SD (Model vs. NC, ^#^ *p* < 0.05, ^###^ *p* < 0.001; other groups vs. Model, * *p* < 0.05, ** *p* < 0.01, *** *p* < 0.001; ns indicates statistically non-significant difference).

**Figure 7:**
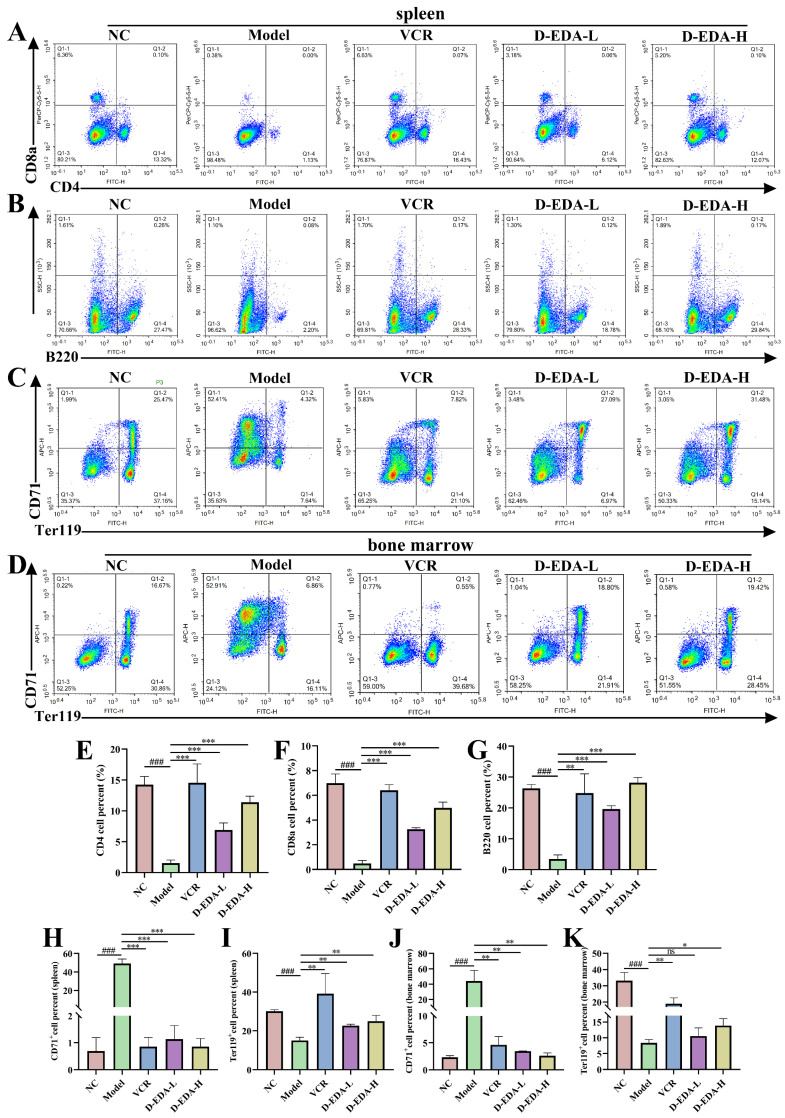
D-EDA reduced the expression of the CD71+ cell population, promoted the expression of the Ter119+ cell population, and activated immune cells in erythroleukemia mice. (**A**,**B**) Changes in CD4, CD8a, and B220 cell populations in mouse spleen. (**C**,**D**) Changes in CD71+ and Ter119+ cell populations in the spleen and bone marrow of mice. (**E**–**G**) Statistical graph of CD4, CD8a, and B220 in mice spleen. (**H**,**I**) Statistical graph of CD71+ and Ter119+ in mice spleen. (**J**,**K**) Statistical graph of CD71+ and Ter119+ in mouse bone marrow. Data are denoted as mean ± SD (*n* = 3. Model vs. NC, ^###^ *p* < 0.001; other groups vs. Model, * *p* < 0.05, ** *p* < 0.01, *** *p* < 0.001; ns indicates statistically non-significant difference).

Cellular experiments have demonstrated the potential effects of D-EDA in HEL cells. To investigate the therapeutic effects of D-EDA in vivo further, we use a mouse model of erythroleukemia induced by Friend murine leukemia virus (F-MuLV). Biochemical analyses of liver and kidney function revealed that D-EDA did not impair these functions, with all parameters remaining within normal ranges (ALT: 10.06–96.47 U/L; AST: 36.31–235.48 U/L; BUN: 10.81–34.74 mg/dL; CREA: 10.91–85.09 μmol/L), indicating that D-EDA is non-toxic to the liver and kidneys in mice (Supplementary Figure S6A–D). Additionally, D-EDA increased hematocrit levels (Figure 6D) and reduced the weights of the spleen, liver, and lungs compared to the Model group, thereby alleviating the burden on these organs in erythroleukemia-affected mice (Figure 6A,C,F,G). Notably, D-EDA did not significantly alter the weights of the heart or kidneys (Figure 6E,H). Histological examination through H&E staining showed that D-EDA mitigated leukemia cell infiltration and restored spleen architecture compared to the Model group (Figure 6I). Furthermore, D-EDA significantly prolonged the survival time of mice with erythroleukemia (Figure 6B). Flow cytometry analysis revealed that D-EDA decreased the population of CD71+ cells and promoted the differentiation of immature erythrocytes into the Ter119+ cell population in both the spleen and bone marrow, thereby alleviating anemia symptoms in the mice (Figure 7C,D,H–K). To assess the immune-modulating effects of D-EDA, flow cytometry was used to evaluate immune cell activation. The results indicated that D-EDA significantly increased the expression of CD4, CD8a, and B220, suggesting enhanced immune cell activation and improved immune function in erythroleukemia mice (Figure 7A,B,E–G).

## 3. Discussion

Acute erythroleukemia (AEL) is a rare form of acute myeloid leukemia (AML) that accounts for less than 5% of cases. It is primarily characterized by impaired erythroid differentiation [26]. Small molecule compounds are still being explored as a new strategy for developing antitumor drugs [27]. In this study, D-EDA was obtained by modifying the structure of EDA. It was found that D-EDA exhibits superior activity compared to EDA in HEL cells. Notably, D-EDA significantly induced apoptosis and blocked cell cycle in HEL cells. Moreover, this study further revealed that D-EDA alleviated the burden on the spleen and liver, restored spleen structure, enhanced the differentiation of Ter119+, CD4+, CD8a+, and B220+ cell populations, and effectively prolonged the survival of erythroleukemia mice in vivo.

Cell death is a crucial process that maintains the balance of cell growth by eliminating unwanted or abnormal cells [28]. It is primarily classified into apoptosis, necrosis, and other forms of cell death based on the underlying mechanisms [29,30]. Among these, apoptosis is a fundamental biological phenomenon characterized by programmed cell death, which can occur under normal physiological and pathological conditions. This genetically regulated process enables cells to terminate their own lives [31] and is primarily triggered by the endogenous mitochondrial pathway and the exogenous death receptor pathway [32,33]. The Bcl-2 family plays a critical role in regulating mitochondrial apoptosis by controlling the permeability of the outer mitochondrial membrane [34]. This study found that D-EDA decreased MMP, induced DNA and mitochondrial damage, down-regulated Bcl-2, and up-regulated the expression of Cleaved Caspase-9, Cleaved Caspase-3, and Cleaved PARP proteins. Inhibition of the caspase cascade reaction using Z-VAD-FMK resulted in a significant reduction in the apoptosis rate and decreased expression levels of these apoptotic proteins in HEL cells [35]. Thus, our findings indicate that the induction of apoptosis in HEL cells by D-EDA depends on the activation of the caspase cascade. Elevated levels of ROS are known to cause mitochondrial DNA damage and dysfunction [22]. Our results indicate that D-EDA increases ROS levels in HEL cells, subsequently leading to apoptosis.

Cell cycle progression and division are fundamental biological processes in animal cells [36]. The cell cycle consists of four distinct phases: G1 (gap phase 1), S (DNA synthesis phase), G2 (gap phase 2), and M (mitosis). Disruption of the cell cycle can lead to uncontrolled cell proliferation, a hallmark of cancer [37,38,39]. In this study, we found that D-EDA effectively blocked the HEL cell cycle in the S phase and down-regulated the expression of CDK2 and cyclinA2 [40]. Additionally, p21, a crucial member of the cyclin-dependent kinase inhibitor family, was up-regulated, highlighting its importance in treating hematological malignancies [41]. Furthermore, c-Myc, an oncoprotein associated with malignant tumor phenotypes [2,42], was down-regulated by D-EDA, suggesting its role in inhibiting cell proliferation in HEL cells.

The discovery of the JAK-STAT signaling pathway, a highly conserved mechanism, has elucidated its critical role in immune responses, cell proliferation, differentiation, and apoptosis [13,43]. This pathway is relevant in various diseases, including cancers, hematological disorders, immune disorders, and inflammatory diseases. JAK2, a member of the JAK family, is primarily associated with treating and preventing hematological disorders [17]. Activation of the JAK-STAT pathway leads to the phosphorylation of STAT family members, which regulates the proliferation and survival of hematopoietic stem cells. The STAT family, particularly STAT3, regulates gene expression upon JAK activation and is associated with the development of several cancers, including leukemia [44,45,46]. While multiple mutations in the JAK-STAT pathway have been implicated in leukemia, the range of approved JAK-STAT inhibitors for leukemia treatment remains limited. Therefore, further research is necessary to explore the complex mechanisms of the JAK-STAT pathway in leukemia and assess the potential impact of new therapeutic agents [47]. In this study, Molecular docking showed that D-EDA binds well to JAK2 with a binding energy of −7.2 kcal/mol. D-EDA significantly down-regulated the expression of phosphorylated JAK2 and STAT3 proteins. Cellular thermal shift assays also showed that D-EDA could bind stably to JAK2 protein. To further investigate whether D-EDA operates through the JAK2-STAT3 pathway in HEL cells, HEL cells were treated with Ruxolitinib, a JAK1/JAK2 inhibitor [48], either alone or in combination with D-EDA. The results demonstrated that D-EDA significantly reduced the protein expression levels of p-JAK2 and p-STAT3 when combined with Ruxolitinib. Furthermore, when combined with Stattic, a STAT3 inhibitor [49], D-EDA further decreased p-STAT3 levels and significantly increased the apoptosis rate, as well as up-regulating the expression of apoptosis-related proteins such as Cleaved Caspase-9, Cleaved Caspase-3, and Cleaved PARP in HEL cells. Above all, this study suggests that D-EDA regulates the JAK2-STAT3 signaling pathway and activates the caspase cascade in HEL cells.

## 4. Materials and Methods

### 4.1. Cell Culture

HEL (human erythroleukemia), K562 (chronic myeloid leukemia), CEM-C1, Jurkat, and CEM-C7H2 (acute lymphoblastic leukemia) cell lines were obtained from the American Type Culture Collection (Manassas, VA, USA). HEL, K562, Jurkat, CEM-C1, and CEM-C7H2 cells were cultured in RPMI-1640 medium with 10% fetal bovine serum (FBS). All cells were maintained at 37 °C in a 5% CO_2_ incubator.

### 4.2. Cell Viability Assays

HEL, K562, Jurkat, CEM-C1, and CEM-C7H2 cells were seeded at 8000 cells/well in 96-well plates. After 4 h, D-EDA and EDA were added at concentrations of 1.25 μM, 2.5 μM, 5 μM, 10 μM, and 20 μM, with a control group (0.1% DMSO group) included. After 12, 24, 48, and 72 h of incubation, absorbance values were measured at 570 nm using a microplate reader (BioTek, Winooski, VT, USA). The half maximal inhibitory concentration (IC_50_) was calculated using SPSS 20.0 (Chicago, IL, USA). Inhibition rate and cell viability were subsequently calculated. The formulas are outlined below: Inhibition rate = (OD of control group − OD of treatment group)/OD of control group × 100%. Cell viability = 1 − inhibition rate × 100%.

### 4.3. Flow Cytometry Analysis of Apoptosis and Cell Cycle

HEL cells (5 × 10^5^) were cultured in 6-well culture plates. After 4 h, D-EDA was added at concentrations of 5 μM, 10 μM, and 20 μM, with a 0.1% DMSO group. For apoptosis detection, the cells were collected at 24 and 48 h and resuspended in 50 μL of 1× binding buffer. They were then stained with 1.5 μL each of PI and Annexin V (BD Biosciences, Franklin Lakes, NJ, USA) for 15 min in the dark, washed with 1× binding buffer, resuspended in 200 μL of 1× binding buffer, and analyzed for apoptosis using flow cytometry (ACEA NovoCyte, San Diego, CA, USA). For the cell cycle analysis, the cells were collected at 24 h and fixed in 500 μL of a 70% ethanol solution at 4 °C for 4 h, followed by overnight storage at −20 °C. The following day, the cells were supplemented with 500 μL of a dye solution prepared by mixing 6 mL of PBS, 300 μL of propidium iodide (PI) (Beyotime, Jiangsu, China), 4 μL of Triton X-100, and 30 μL of RNase A for 30 min in the dark; they were then resuspended in 200 μL of PBS, filtered, and analyzed using flow cytometry to assess cell cycle distribution.

### 4.4. Hoechst 33258 Staining

HEL cells (5 × 10^5^) were inoculated in 6-well culture plates and treated with D-EDA (5, 10, and 20 μM) for 48 h. The cells were collected, and 300 μL of Hoechst 33258 (Beyotime, Jiangsu, China) solution was added for 15 min at 37 °C in the dark. Following incubation, 50 μL of the cell suspension was transferred onto a slide using a pipette, covered with a coverslip, and observed under an inverted fluorescence microscope.

### 4.5. Mitochondrial Membrane Potential (MMP) Assay

HEL cells (5 × 10^5^) were cultured in 6-well plates and treated with D-EDA (5, 10, and 20 μM) for 24 h. The cells were collected and resuspended in 500 μL of FBS-free RPMI-1640. Next, 500 μL of the JC-1 staining working solution (Beyotime, Jiangsu, China) was added for 20 min at 37 °C in the dark. After incubation, the cells were washed with 1× JC-1 staining buffer, and the supernatant was discarded. The cells were then resuspended in 200 μL of 1× JC-1 staining buffer. Finally, the impact of D-EDA on the MMP of HEL cells was assessed using flow cytometry and fluorescence microscopy.

### 4.6. Reactive Oxygen Species (ROS) Assay

HEL cells (5 × 10^5^) were seeded in 6-well culture plates and treated with D-EDA (5, 10, and 20 μM) for 24 h. The cells were collected, and 500 μL of diluted DCFH-DA working solution (Solarbio, Beijing, China) was incubated for 20 min at 37 °C in the dark. The effect of D-EDA on ROS in HEL cells was then detected using flow cytometry and fluorescence inversion microscopy.

### 4.7. Western Blotting

HEL cells (15 × 10^5^) were inoculated in 60 mm cell culture dishes and treated with D-EDA (5, 10, and 20 μM) for 24 h. The cells were collected, and an appropriate volume of immunoprecipitation (IP) lysis buffer containing phenylmethanesulfonyl fluoride (PMSF) at a 100:1 ratio was added based on the cell pellet for 40 min on ice, followed by centrifugation at 12,000 rpm for 15 min. The supernatant was collected, and the protein concentration was determined using a BCA protein assay kit (Solarbio, Beijing, China). The protein extract was mixed with 5 × loading buffer in a 1:4 ratio, denatured at 98 °C for 5 min, and stored at −20 °C. Proteins were separated using SDS-PAGE electrophoresis at 120 V for 120 min, followed by wet transfer to a PVDF membrane (0.22 µM, Bio-Rad, Hercules, CA, USA) at 220 mA for 120 min. Subsequently, the membrane was blocked with 5% milk for 1.5 h and incubated at 4 °C overnight. The next day, it was washed with 1 × TBS and incubated with secondary antibodies in the dark for 2 h. Finally, the membrane was developed using the Odyssey Platform (LI-COR Biosciences, Lincoln, NE, USA) to detect changes in the expression of target proteins.

### 4.8. Molecular Docking and Inhibitor Treatment

The 3D structure of D-EDA (PubChem CID: 285802) was obtained from the PubChem database (https://pubchem.ncbi.nlm.nih.gov accessed on 18 September 2024). The protein structure of JAK2 (PDB: 7REK) was sourced from the Protein Data Bank (https://www.rcsb.org/ accessed on 18 September 2024). D-EDA was docked to JAK2 using AutoDock Vina (Version 1.1.2) [50]. The result of the docking simulation was visualized by PyMOL (Version 3.1.0a0). HEL cells were further treated with a JAK2 inhibitor (Ruxolitinib) and a STAT3 (Stattic) inhibitor for 24 h. The effects of Ruxolitinib (5 nM) alone or in combination with D-EDA (10 μM) on the JAK2, p-JAK2, STAT3, and p-STAT3 proteins. The effects of static (4 μM) alone or in combination with D-EDA (10 μM) on apoptosis were examined by flow cytometry and further analyzed the effects on STAT3, p-STAT3, Caspase-9, Cleaved Caspase-9, Caspase-3, Cleaved Caspase-3, PARP, and Cleaved PARP protein expression by Western blotting.

### 4.9. Cellular Thermal Shift Assay (CETSA)

HEL cells (2 × 10^6^) were seeded in 60 mm cell culture dishes and treated with D-EDA (80 μM) for 2 h. Cells were collected, and the proteins were extracted. The protein extracts were then heated at gradients of 37 °C, 40 °C, 43 °C, 46 °C, 49 °C, 52 °C, 55 °C, 58 °C, and 61 °C for 3 min each. The extracts were mixed with a 5 × loading buffer in a 1:4 ratio and denatured at 98 °C for 5 min. Western blotting was subsequently performed to assess the impact of D-EDA on JAK2 protein expression.

### 4.10. In Vivo Experiment

NIH3T3 cells expressing Friend murine leukemia virus (F-MuLV) clone 57 vectors were kindly donated by Prof. Ben-David Yaacov, Natural Products Research Center of Guizhou Province. BALB/c mice (Tengxin, Chongqing, China) were modeled by intraperitoneal injection of Friend murine leukemia virus (F-MuLV) within one day of birth [51]. After 5 weeks of viral infection, mice were divided into 5 groups: Normal control (NC), Model, Positive drug group (VCR: 0.3 mg/kg), D-EDA low-dose group (D-EDA-L: 2.5 mg/kg), and D-EDA high-dose group (D-EDA-H: 5 mg/kg), 12 mice per group, an equal number of males and females. The mice were administered D-EDA every two days and VCR once a week by intraperitoneal injection. After 2 weeks of treatment, the blood volume ratio of mice was measured and the organs were weighed. H&E pathology sections of the spleen were examined. Serum was separated for liver function aspartate aminotransferase (AST), alanine aminotransferase (ALT), kidney function blood urea nitrogen (BUN), and creatinine (CREA). In addition, flow cytometry was utilized to detect changes in CD71 and Ter119 cell populations in single-cell mixtures from bone marrow and spleen. CD4, CD8a, and B220 immune system-associated markers in the spleen were examined by flow cytometry [52]. Finally, the remaining mice were used to evaluate survival curves.

### 4.11. Statistical Analysis

The experiments were independently replicated at least three times. The experimental data are presented as the mean ± standard deviation (mean ± SD). Statistical analyses were conducted using a two-tailed Student’s *t*-test or one-way ANOVA with Tukey’s post-hoc test. *p* < 0.05 was considered statistically significant. Additionally, data analysis and visualization were performed using GraphPad Prism 8.0 (GraphPad Software, La Jolla, CA, USA).

## 5. Conclusions

**Figure 8:**
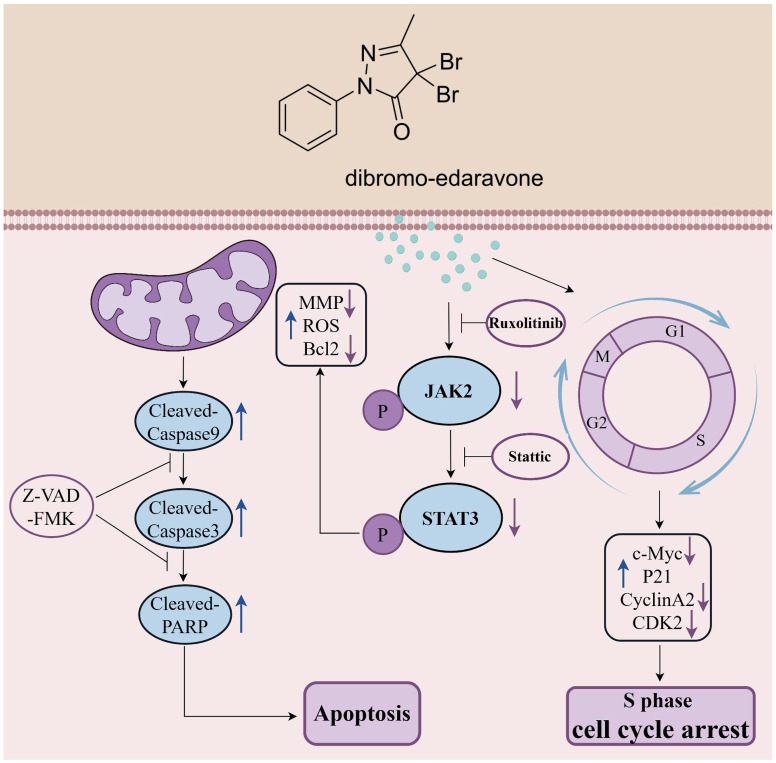
Molecular mechanism of D-EDA on anti-erythroleukemia (The red arrows represent down-regulation, and the blue arrows represent up-regulation).

In summary, the small molecule compound D-EDA exhibited anti-erythroleukemia effects in vivo and in vitro. We found that D-EDA demonstrated significant anti-leukemic activity by inhibiting cell viability, inducing apoptosis via activation of the caspase cascade, causing cell cycle arrest, and modulating the JAK2-STAT3 signaling pathway in HEL cells (Figure 8). Furthermore, D-EDA promoted the differentiation of mature erythrocytes, activated immune cells, and effectively prolonged the survival time in erythroleukemia mice. These findings suggest that D-EDA holds significant promise as a therapeutic agent for the treatment of acute erythroleukemia.

## Data Availability

The data provided in this study are available for reference.

## Supplementary Materials

The following supporting information can be downloaded at: https://www.mdpi.com/article/10.3390/ijms26094000/s1.
