# Supplementary material for: Dibromo-Edaravone Induces Anti-Erythroleukemia Effects via the JAK2-STAT3 Signaling Pathway
Source: Int J Mol Sci. 2025 Apr 23;26(9):4000. doi: 10.3390/ijms26094000 (PMC12071273; doi:10.3390/ijms26094000)
Supplement: Supplementary file 1 [file ijms-26-04000-s001.zip › ijms-3438857-supplementary.pdf]

Supplementary Materials

1. Materials and Methods

1.1 Reagents

RPMI-1640 and DMEM media, as well as fetal bovine serum (FBS) (Gibco, USA), were used in cell culture, along with a 100× penicillin-streptomycin mixture (Hyclone, USA). The Annexin V FITC/PI apoptosis kit was obtained from BD Pharmingen (USA), while RNase A was sourced from TaKaRa (China). Other reagents, including Triton X-100, reactive oxygen species (ROS) assay kits, dimethyl sulfoxide (DMSO), bovine serum albumin (BSA), thiazolyl blue tetrazolium bromide (MTT), and BCA protein assay kits, were purchased from Solarbio Life Sciences (Beijing, China). Modified Giemsa Staining Solution, Propidium iodide (PI), mitochondrial membrane potential (MMP) assay kits with JC-1, and Hoechst 33258 were acquired from Beyotime Biotechnology (Shanghai, China). Stattic and Ruxolitinib were purchased from MCE. Vincristine (VCR) was purchased from Yifan Pharmaceutical Company (Anhui, China). Anti-Mouse Ter119 (FITC), CD4 (FITC), CD8a (PerCP-Cy5.5), and B220 (FITC) were purchased from BD Pharmingen (USA). Anti-Mouse CD71 (APC) was procured from Thermo Fisher (USA).

For antibody-based experiments, STAT3 (ab119352) and p-STAT3 (ab76315) antibodies were sourced from Abcam (UK), and JAK2 (#3230T), c-Myc (#5605T), CyclinA2 (#67955T), CDK2 (#2546T), p21 (#2947S), Bcl-2 (#4223T), Caspase-3 (#14220T), Caspase-9 (#9502T), PARP (#9532T), and anti-rabbit IgG (H + L) DyLight 800 4X PEG conjugate (#5151S) were from Cell Signaling Technology (CST, USA). Additionally, p-JAK2 (381556) was procured from ZEN-BIO (China) and GAPDH (AF7021) from Affinity Biosciences (USA).

1.2 Observation of Cell Morphology

HEL cells (5×10^5) were seeded in 6-well culture plates. After 4 h, D-EDA was added at concentrations of 5 μM, 10 μM, and 20 μM, with a 0.1% DMSO group. A

microscope observed the effects of D-EDA on HEL cell morphology at 24 and 48 h.

1.3 Giemsa Staining

HEL cells (5×10^5) were seeded in 6-well culture plates and treated with D-EDA (5, 10, and 20 μM) for 24 h. Cells were collected and resuspended with 70% ethanol. A total of 80 microliters of cell suspension was added to one end of the slide, and then the slides were pushed evenly, fixed for 10 minutes, dried in the fume hood, and stained with 1×Giemsa staining solution for 45 minutes. At the end of the staining, the cells were washed well with double-distilled water from one side, dried, and photographed for observation under an inverted microscope.

1.4 Quantitative Real-Time PCR (qPCR)

HEL cells (2×10^6) were seeded in 60 mm cell culture dishes and treated with D-EDA (5, 10, and 20 μM) for 24 h. Cells were collected, and total RNA was extracted using the TRIzol reagent. The RNA was then reverse-transcribed to cDNA using the PrimeScript RT reagent Kit with the gDNA Eraser (TaKaRa, Beijing, China). The mRNA levels were quantified using specific primers and Fast Start Universal SYBR Green Master (Roche) in a StepOne Plus thermal cycler (Applied Biosystems, Carlsbad, CA, USA). GAPDH was used as an endogenous DMSO. Sequences of JAK2 primers (Sangon Biotech, Shanghai, China) were as follows:

Sense: 5’-ATCCACCCAACCATGTCTTCC-3’

Antisense: 5’-ATTCCATGCCGATAGGCTCTG-3’

2. Results

2.1 Chemistry

NBS (378 mg, 2.1 mmol) was added to a solution of edaravone (174 mg, 1.0 mmol) in dichloromethane (20 mL). The reaction mixture was stirred at room temperature for 1 h. The solution was washed with H_2O (20 mL), brine (20 mL), and concentrated under reduced pressure. The residue was purified using column chromatography on silica gel (10% EtOAc in hexane as eluant) to obtain 318 mg dibromo-edaravone (D-EDA) as a yellow solid with a 96% yield.

D-EDA: m.p. 74.6-76.9 °C; ^{1}H NMR (600 MHz, $CDCl_3$) δ 2.48 (s, 3H) 7.29 (d, *J* = 8.1 Hz, 1H) 7.46 (t, *J* = 7.9 Hz, 2H) 7.90 (d, *J* = 8.3 Hz, 2H); ^{13}C NMR (150 MHz, $CDCl_3$) δ 13.1, 46.0, 118.8, 126.0, 129.0, 136.9, 156.0, 165.2; The NMR data is consistent with the literature values [24]. Purity > 98%.

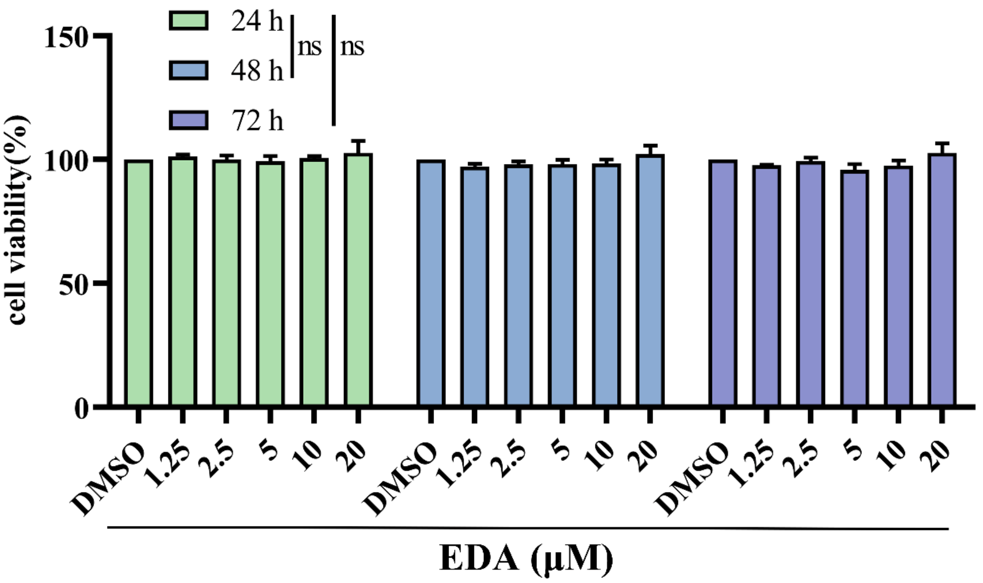

Figure S1. EDA has no effect on HEL cells. The survival rate of EDA-affected HEL cells at 24, 48, and 72 h. Data are denoted as mean ± SD (n = 3, $P > 0.05$, ns indicates no significant differences vs. 24 h).

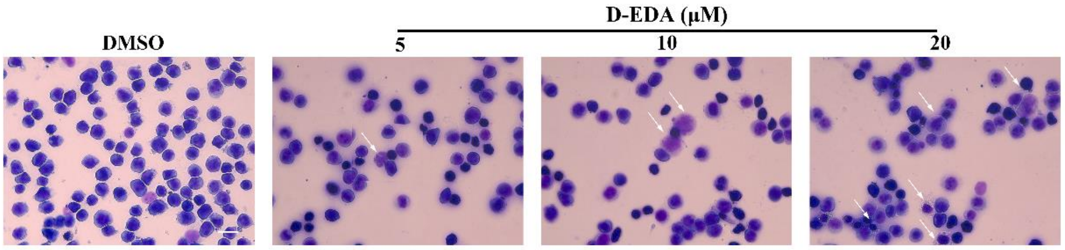

Figure S2. The changes of apoptosis in HEL cells treated with D-EDA for 24 h using Giemsa staining (Magnification: 400×, Scale bar: 50μm).

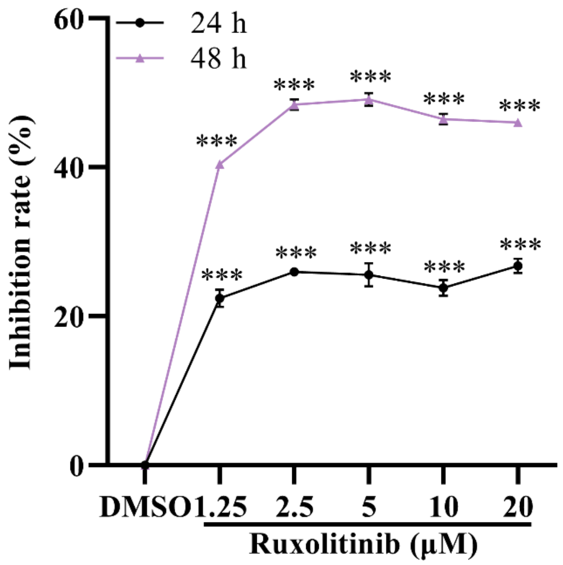

Figure S3. Ruxolitinib inhibits the viability of HEL cells. The inhibition rate of Ruxolitinib in HEL cells is 24 h and 48 h. Data are denoted as mean ± SD (n = 3. ***P < 0.001 vs. the control group).

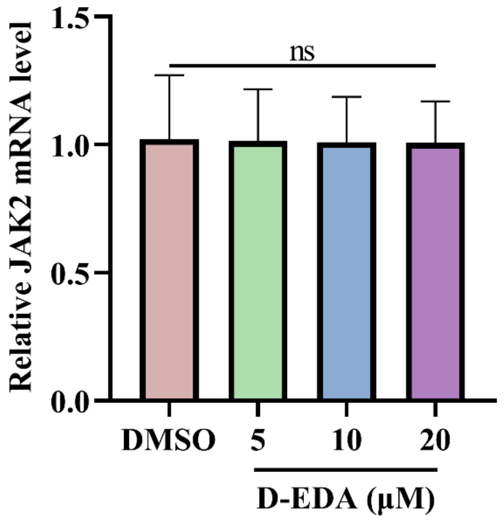

Figure S4. D-EDA does not affect JAK2 mRNA level in HEL cells. The expression of JAK2 at the mRNA level was assessed with D-EDA treatment on HEL cells for 24 h. Data are denoted as mean ± SD (n = 3. P > 0.05, ns indicates no significant differences vs. the control group).

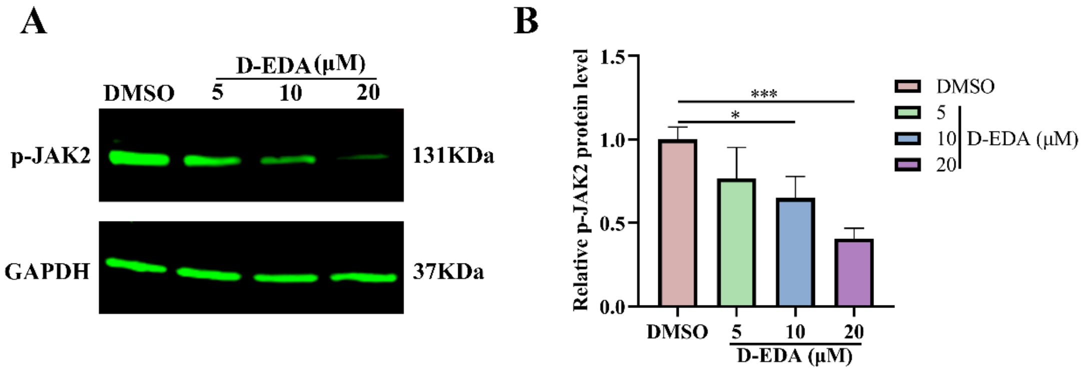

Figure S5. D-EDA downregulates p-JAK2 protein in K562 cells. (A) Effects of D-EDA on K562 cells for 24 h on p-JAK2 protein. (B) Statistical graph of p-JAK2 protein. Data are denoted as mean ± SD (n = 3. *$P < 0.05$, ***$P < 0.001$ vs. the control group).

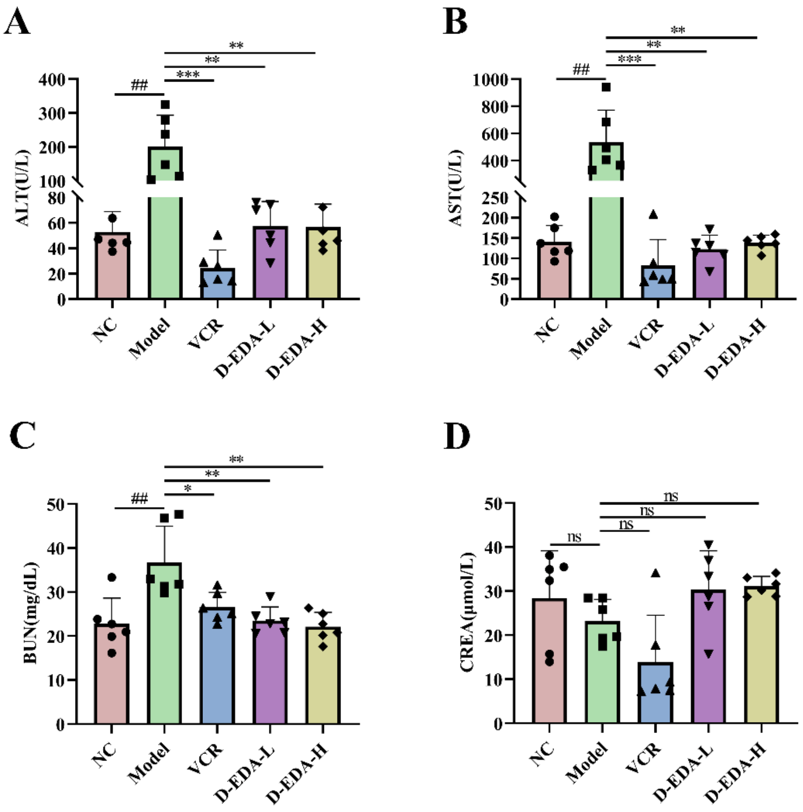

Figure S6. D-EDA does not affect liver and kidney function in mice. (A) Alanine aminotransferase (ALT). (B) Aspartate aminotransferase (AST). (C) Blood urea nitrogen (BUN). (D) Creatinine (CREA). Data are denoted as mean ± SD (n = 6. Model vs. NC, $^{\#\#}P < 0.01$; other groups vs. Model, *$P < 0.05$, **$P < 0.01$, ***$P < 0.001$; ns indicates statistically non-significant difference).
